# Effect of mycalolides isolated from a marine sponge *Mycale* aff. *nullarosette* on actin in living cells

**DOI:** 10.1038/s41598-019-44036-2

**Published:** 2019-05-17

**Authors:** Yoko Hayashi-Takanaka, Yuto Kina, Fumiaki Nakamura, Shota Yamazaki, Masahiko Harata, Rob W. M. van Soest, Hiroshi Kimura, Yoichi Nakao

**Affiliations:** 1 Graduate School of Bioscience and Biotechnology, Tokyo Institute of Technology, Yokohama, 226-8503 Japan; 2 Graduate School of Frontier Biosciences, Osaka University, Suita, 565-0871 Japan; 3 Graduate School of Advanced Science and Engineering, Waseda University, Tokyo, 169-8555 Japan; 4 Graduate School of Agricultural Science, Tohoku University, Sendai, 980-0845 Japan; 5 Naturalis Biodiversity Center, 2300 RA Leiden, The Netherlands; 6 Cell Biology Center, Institute of Innovative Research, Tokyo Institute of Technology, Yokohama, 226-8503 Japan; 7 Present Address: RIKEN Center for Advanced Photonics, Sendai, 980-0845 Japan

**Keywords:** Cellular imaging, Actin, Target identification

## Abstract

Discovery of novel bioactive compounds is important not only for therapeutic purposes but also for understanding the mechanisms of biological processes. To screen bioactive compounds that affect nuclear morphology in marine organism extracts, we employed a microscopy-based assay using DNA staining of human cancer cells. A crude extract from a marine sponge *Mycale* aff*. nullarosette*, collected from the east coast of Japan, induced cellular binucleation. Fractionation of the extract led to the isolation of mycalolides A and B, and 38-hydroxymycalolide B as the active components. Mycalolides have been identified as marine toxins that induce depolymerization of the actin filament. Live cell imaging revealed that low concentrations of mycalolide A produce binucleated cells by inhibiting the completion of cytokinesis. At higher concentrations, however, mycalolide A causes immediate disruption of actin filaments and changes in cell morphology, yielding rounded cells. These results suggest that the completion of cytokinesis is a process requiring high actin polymerization activity. Furthermore, luciferase reporter assays with mycalolide A treatments support the view that the level of globular actin can affect transcription of a serum response gene.

## Introduction

Marine invertebrates, including marine sponges, are a good source of natural products with potent bioactivities. For example, some protein phosphatase inhibitors such as okadaic acid and calyculin A that serve as biological probes for studying signal transduction mechanisms^1^ and compounds that target cytoskeletal filaments, such as actin-filaments and microtubule-filaments, have been isolated from marine organisms^2^.

Actin is the most abundant protein in typical eukaryotic cells and participates in many fundamental cellular processes including muscle contraction, cell motility, cell division and cytokinesis. Moreover, actin is involved in the transcriptional regulation of a subset of genes^3,4^. Many of these processes are controlled through the balance between actin polymerization (to form filamentous actin, or F-actin) and depolymerization (to dissociate into monomeric globular actin, or G-actin)^5,6^. Such a polymerization-depolymerization balance of actin can be disturbed by actin-targeting small compounds. The latrunculins, which were initially isolated from the Red Sea sponge *Negombata* (*Latrunculia*) *magnifica*^7^, bind to monomeric G-actin and inhibit its polymerization. In contrast, jusplakinolide, isolated from the Indo-Pacific sponge *Jaspis johnstoni*, promotes actin polymerization and binds to F-actin^8^. Mycalolides that are composed of a macrocyclic ring containing three adjacent oxazoles were identified as marine toxins that induce actin depolymerization by binding to G-actin^9–11^. The macrocyclic ring is attached to a long acyclic side chain with minor variations. The actin-binding property of mycalolide B has been characterized extensively *in vitro* but the binding property of mycalolides in living cells has been only slightly described^10^. Mycalolide B has been recently shown to disrupt dynactin complexes and inhibit neuronal retrograde transport at a relatively high concentration^12^.

Here, we describe the isolation of three mycalolides from the marine sponge *Mycale* aff. *nullarosette* collected at the coast of Miyagi, Japan, using a cell-based microscopy assay. We initially detected, via DNA staining, the induction of binucleated cells by the crude marine sponge extract. During fractionation of the active components, we found that the fractions that induced binucleation were also associated with actin depolymerizing activity, as revealed by F-actin staining using rhodamine-conjugated phalloidin. Three fractions that produced binucleation and actin depolymerization in cells were found to contain mycalolides A, B, and 38-hydroxymycalolide B. We then employed live-cell imaging to directly monitor the effect on actin filaments and cell morphology of the fraction containing mycalolide A. When cells were incubated with the fraction at low concentrations, cells entered into mitosis, but harbored two daughter nuclei. At higher concentrations, actin fibers were immediately disrupted, causing the loss of cellular tension. These results are consistent with the biochemical property observed *in vitro* for mycalolides that bind to F-actin to promote depolymerization. In addition, the luciferase reporter assay suggests that mycalolide A can bind to nuclear actin and affect transcription from a serum response gene.

## Materials and Methods

### Animal material

*Mycale* aff. *nullarosette* was collected at a depth of 2 m off the coast of Sameura bay, Miyagi, Japan (38°22′N; 141°29′E) in September 2007 (Supplementary Fig. S1A). Another *Mycale* was collected at the depth of 15 m in the Ikara Straits, Kagoshima, Japan (32°22′N; 130°19′E) in August 2013. The specimens were immediately frozen and kept at −30 °C or −25 °C until processed.

### Extraction and isolation

Mycalolides from Miyagi *Mycale* were isolated as described below. The frozen material (232 g) was extracted with methanol (MeOH) at room temperature. The crude methanolic extract was concentrated and partitioned between H_2_O and CHCl_3_. The aqueous phase was extracted with *n*-BuOH and the *n*-BuOH phase was combined with the CHCl_3_ phase. The combined extract was subjected to the modified Kupchan’s procedure^13^. The sample was partitioned between *n*-hexane and MeOH/H_2_O (9:1), and water was added to the aqueous MeOH layer to adjust the water content to 40%, which then was extracted with CHCl_3_.

The obtained CHCl_3_ layer was concentrated and applied to an ODS flash column (ϕ3.5 × 6 cm) which was eluted in a stepwise manner with MeOH/H_2_O (5:5) and (7:3), acetonitrile (MeCN)/H_2_O (7:3) and (17:3), MeOH, and CHCl_3_/MeOH/H_2_O (6:4:1). The fractions were subjected to a cell-based assay. The active MeOH/H_2_O (7:3) eluting fraction, which induces the binucleation of MDA-MB-231 cells (at a concentration of less than 8 ng/ml), was separated by reversed phase ODS HPLC (COSMOSIL 5C_18_ AR-II; 65% MeOH) to yield mycalolide B (23.9 mg) as well as a mixture of mycalolide A and 38-hydroxymycalolide B. The latter mixture (32.2 mg) was further fractionated through a reversed phase HPLC (Develosil C_30_ UG-5; 75% MeOH) to yield mycalolide A (11.5 mg) and 38-hydroxymycalolide B (12.6 mg).

Mycalolides were also isolated from Kagoshima *Mycale* (1180 g) using essentially the same procedure as above, except that a reversed phase ODS HPLC fraction containing mycalolides was further fractionated through a reversed phase HPLC (CAPCELL PAK UG120; 70% MeOH) to yield mycalolide A (13.4 mg).

NMR spectra of mycalolides were acquired using an Avance 400 MHz spectrometer (Bruker). ^1^H NMR chemical shifts were referenced to the solvent peak at 3.31 ppm for CHD_2_OD. Liquid chromatography followed by mass spectrometry (LC-MS) data were obtained using a TripleTOF 4600 mass spectrometer in the positive mode (AB Sciex), equipped with an ultra-fast liquid chromatography system (Shimadzu) using C_18_ column (COSMOSIL 3C_18_-AR-II [2.0 ID × 50 mm]; flow rate 0.3 mL/min; gradient elution from 5 to 100% MeCN with 0.1% HCOOH over 15 min). The structures of mycalolide A, mycalolide B, and 38-hydroxymycalolide B were identified by comparison of the NMR spectra and MS data with the literature^10,11,14^.

### Cell-based screening

MDA-MB-231 human breast cancer cells^15^ and HeLa human cervical cancer cells^16^ were routinely grown in Dulbecco’s modified Eagle’s medium (DMEM; Nacalai Tesque) supplemented with antibiotics (10 U/ml penicillin, 50 μg/ml streptomycin) and 10% fetal calf serum (FCS). For screening, cells were plated into 96-well glass-bottom plates (Iwaki) in DMEM containing 10 μg/ml extracts (2–4 × 10^3^ cells/100 μl in each well), incubated for 20 hr in a CO_2_ incubator, and fixed with 4% formaldehyde (Electron Microscopy Sciences) in 250 mM Hepes-NaOH (pH 7.4; Wako Pure Chemical Industries) for 10 min at room temperature. After washing three times with phosphate-buffered saline (PBS; Wako Pure Chemical Industries), cells were stained with 10 ng/ml Hoechst 33342 (Nacalai Tesque) in PBS containing 0.5% Triton X-100 (Nacalai Tesque) for 2 hr at room temperature^15^. In some cases, cells were also treated with 1 μM nocodazole (Nacalai Tesque) for 1 hr before fixation, and then stained with 20 ng/ml rhodamine-conjugated phalloidin (Sigma-Aldrich), 1:1000 mouse anti-tubulin antibody (Sigma-Aldrich) together with Hoechst 33342.

To compare the rate of binuclear cell formation, cells were plated at different densities in 12-well plates, and 4 hr later, purified mycalolide A, cytochalasin D (Wako Pure Chemical Industries) and latrunculin B (Wako Pure Chemical Industries) were added. Cells were cultured for 20 hr, before fixation and staining with Hoechst 33342 and rhodamine-phalloidin.

Fluorescence images of cells in individual wells were collected using an inverted microscope (Ti-E, Nikon) equipped with a motorized XY-stage (Nikon) with a PlanApo 20× dry objective lens (NA = 0.6), combined with an electron multiplying charge coupled device (EM-CCD; iXon+; Andor; normal mode; gain ×5.1), under the operation of NIS Element ver 3.0 (Nikon).

### Live cell microscopy

For live cell imaging, HeLa human cervical cancer cells were plated on a glass-bottom dish (Mat-Tek) and the medium was replaced by phenol red-free DMEM (Nacalai Tesque) supplemented with antibiotics and 10% FCS. Rhodamine-conjugated actin (1 mg/ml; 3 μl; Cytoskeleton) was introduced into cells using glass beads^17^. Phase contrast and fluorescence images were captured using an inverted microscope (Ti-E; Nikon), featuring a culture system (Tokai Hit) at 37 °C under 5% CO_2_, with a PlanApo VC 100× (NA = 1.4) oil-immersion objective lens, using an EM-CCD (iXon+; Andor; normal mode; gain × 5.1) with filter sets (Semrock; DAPI-1160 for Hoechst 33342 and LF561-A for rhodamine) and the exposure period set to 200 ms. A 75 W Xenon lamp was used as a fluorescence light source and attenuated through neutral-density and 440 nm long-pass filters to achieve a light intensity of 6–10 μW at the specimen. Phase-contrast images were collected (200 ms) using an external phase ring.

### Dual-luciferase reporter assay

The dual-luciferase reporter system (Promega) was used; the pGL4.34[luc2P/SRF-RE/Hygro] harbors a luciferase reporter gene under a promoter containing SRF response elements, and the pGL4.74[hRluc/TK] expresses Renilla luciferase for normalization of transfection efficiency. HeLa cells in 24-well plates (4.0 × 10^4^ cells/cm^2^) were transfected using Lipofectamine 2000 (Thermo Fisher Scientific; 0.8 μl) with pGL4.34[luc2P/SRF-RE/Hygro] (400 ng/well) and pGL4.74[hRluc/TK] (8 ng/well). Twenty four hr after transfection, cells were treated with mycalolide A for 3 hr, and luminescence produced by luciferase catalytic activity was measured using the Dual-glo luciferase assay system (Promega) with a GloMax 96 well Microplate Luminometer (Promega) according to the manufacturer’s instructions. Statistical analysis was performed using the two-sided Student’s t-test (Microsoft Excel 2013).

## Results and Discussion

### Purification of mycalolides in cell based assays

**Figure 1:**
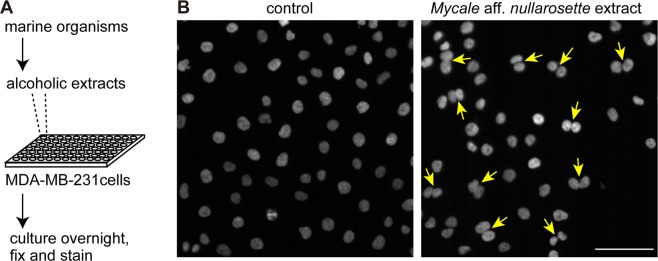
The initial screening using a cell-based assay. (**A**) The scheme for the screening. MDA-MB-231 cells were grown for 20 hr in the presence or absence of 10 μg/ml alcoholic extracts from marine organisms. The cells were fixed, and stained with Hoechst 33342. (**B**) Formation of binucleated cells. Arrows indicate the binucleated cells observed in 10 μg/ml crude extract of marine sponge *Mycale* aff. *nullarosette*. The arrows indicate the cells harboring two daughter nuclei. Bar, 100 μm.

A cell-based assay monitored by a motorized fluorescence microscope equipped with an EM-CCD was used to screen bioactive compounds from marine organisms. MDA-MB-231 breast cancer cells were grown in 96-well glass-bottom plates and cultured for ~20 hr with the organic compounds extracted from the marine organisms. Cells were fixed and incubated with a DNA-staining dye, Hoechst 33342, to evaluate the effects of the compounds on the nuclear morphology (Fig. 1A). An extract from *Mycale* aff*. nullarosette*, collected at Miyagi prefecture, the North-Eastern coast of Japan (Supplementary Fig. S1A), was found to promote the production of cells harboring two nuclei, or binucleated cells (Fig. 1B).

**Figure 2:**
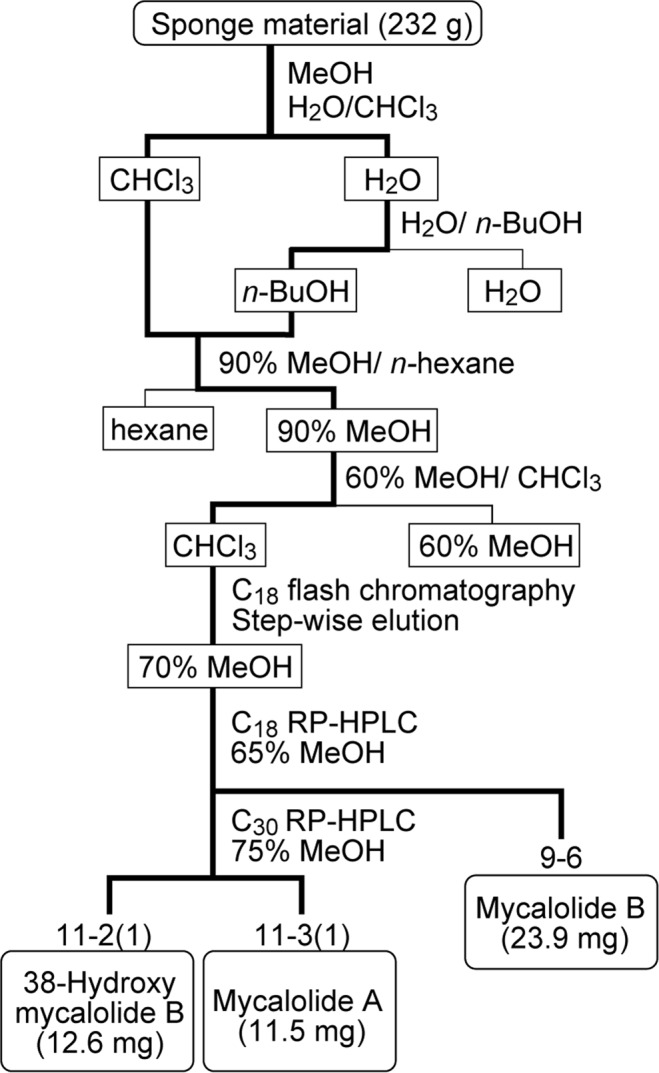
Purification scheme of mycalolides from marine sponge *Mycale* aff. *nullarosette*.

**Figure 3:**
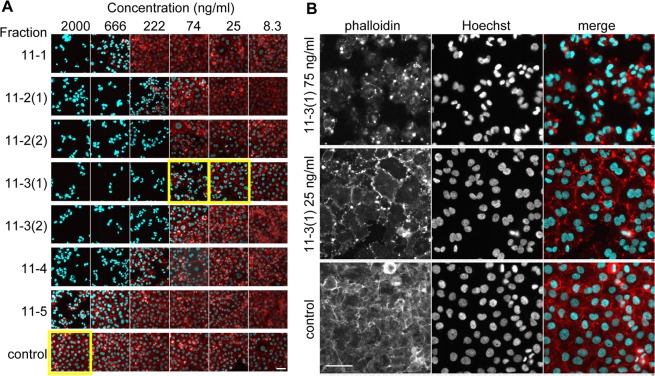
The second screening using Hoechst 33342 and rhodamine-conjugated phalloidin. Cells were grown for 20 hr in the presence of column fractions. The cells were fixed, and stained with Hoechst 33342 (cyan) and rhodamine-phalloidin (red). (**A**) Low magnification views. (**B**) High magnification views of samples indicated by yellow in (**A**). Single-color and merged images are shown. Bars, 100 μm.

**Figure 4:**
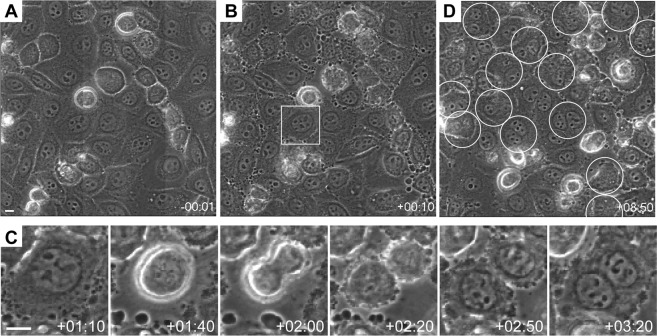
Effects of the fraction 11-3(1) (82% mycalolide A) on cytokinesis. (**A–D**) Live cell imaging. Phase-contrast images of HeLa cells were collected every 10 min, and 10 ng/ml mycalolide A was added at time point 00:00 (hh:mm). Large images are composited from 9 (3 × 3) original images next to each other. Bar, 10 μm. (**A**,**B**) Images just before (**A**) and after (**B**) the addition of mycalolide A. (**C**) Time-lapse images of a cell (indicated in (**B**)) that went into mitosis. (**D**) Cells at 8:50 after mycalolide A addition. Cells that failed to complete cytokinesis and harbored two nuclei are indicated by circles.

**Figure 5:**
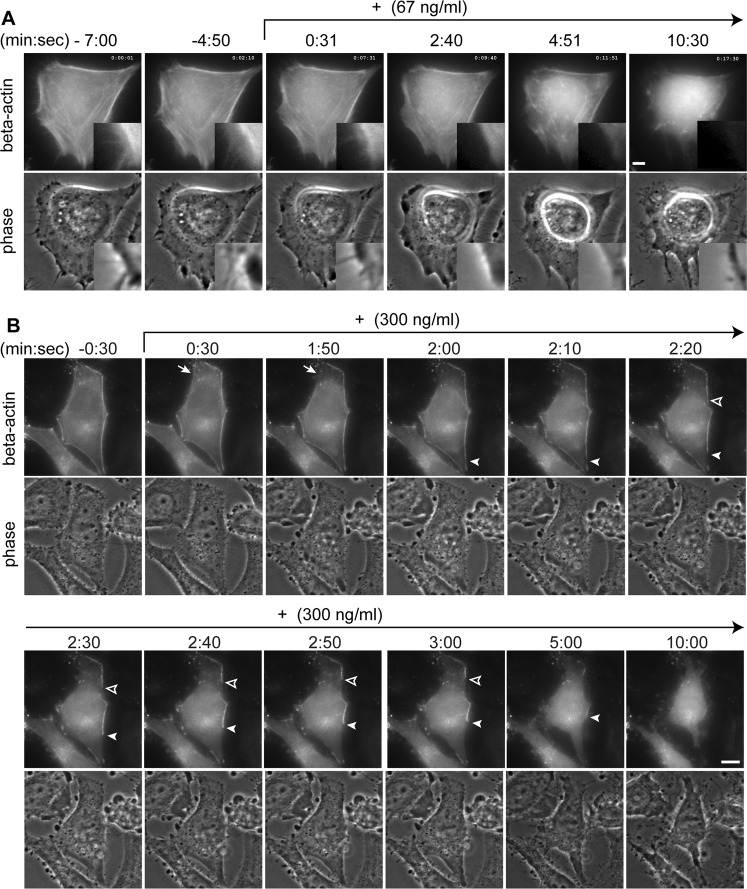
Effects of the fraction 11-3(1) (82% mycalolide A) on actin filaments in living cells. Cells grown on a glass-bottom dish were loaded with rhodamine-actin, and fluorescence and phase contrast images were collected every 5 sec. The fraction 11-3(1) (82% mycalolide A) was added at a final concentration of 67 ng/ml (**A**) and 300 ng/ml (**B**). Insets show higher magnified views of the indicated area. Bars, 10 μm.

**Figure 6:**
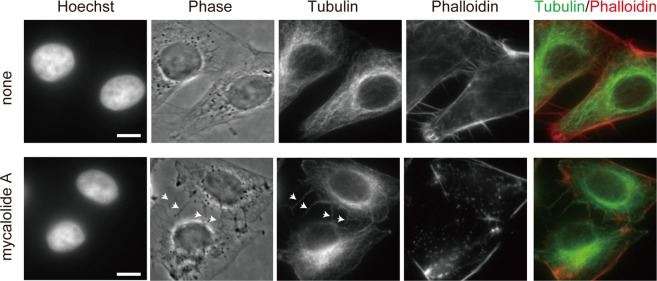
Distribution of tubulin in cells treated with the fraction 11-3(1) (82% mycalolide A). HeLa cells were treated with or without 67 ng/ml. The fraction 11-3(1) (82% mycalolide A) for 10 min. After fixation, cells were stained with Hoechst 33342, anti-tubulin, and phalloidin. Spikes appeared in mycalolide A-treated cells containing tubulin but not F-actin (arrowheads), unlike filopodia in untreated cells. Bars, 10 μm.

**Figure 7:**
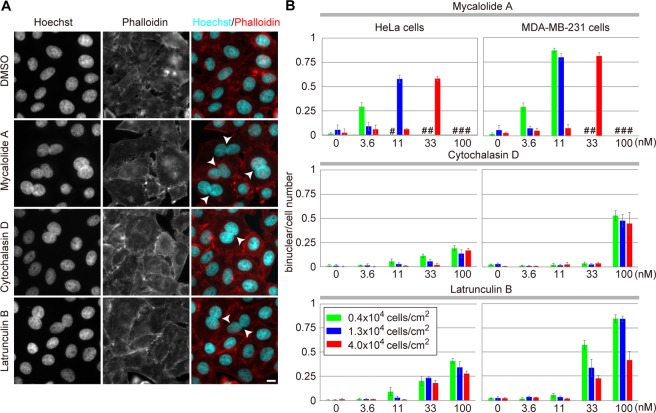
The rates of binucleated cell formation by actin polymerization inhibitors. HeLa cells and MDA-MB-231 cells were treated for 20 hr with mycalolide A (92% purity), cytochalasin D, and latrunculin B at the indicated inhibitor concentration and cell density. The cells were fixed, and stained with Hoechst 33342 and rhodamine-phalloidin. (**A**) Representative images of HeLa cells (starting cell numbers were 13 × 10^3^ cells/cm^2^) treated with or without 33 nM of actin inhibitors are shown. Binucleated cells are indicated by arrowheads. Bar, 10 μm. (**B**) The proportion of binucleated cells. Using images like those presented in (**A**), more than 100 cells were counted in each condition and the rate of binucleated cells were calculated. Means ± S.D. from three biological replicates are shown. In some conditions (indicated by #), cell number was too low to be counted as a result of cell death.

**Figure 8:**
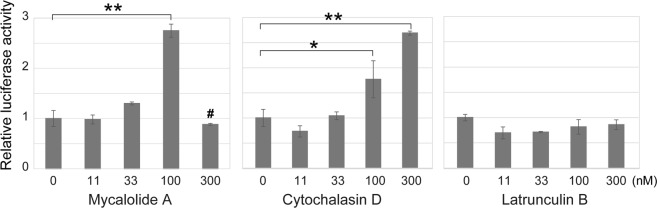
Effects of mycalolide A on SRF-responsive transcription by the luciferase reporter assay. SRF-responsive luciferase activity was measured in HeLa cells treated with the indicated concentrations of mycalolide A (92% purity), cytochalasin D, and latrunculin B for 3 hr. After normalizing the activities with co-transfected Renilla luciferase, the relative values to that in cells treated with DMSO (the solvent) were obtained. Data are expressed as means ± SD (from 3 independent experiments), where asterisks indicate a statistically significant difference (**p ≤ 0.01; *p ≤ 0.05). In 300 nM mycalolide A, many dead cells were observed and so the value at this condition (#) should not be directly compared with the others.

To purify the active components that cause binucleation, the extract was fractionated by solvent partitioning and column chromatography (Fig. 2). Since cellular binucleation is typically observed in agents that affect actin-filament turnover^18–20^, F-actin was also stained with rhodamine-conjugated phalloidin for subsequent assays. The F-actin formation was indeed disturbed by the active fractions (Fig. 3). For example, F-actin disappeared when cells were incubated at higher concentrations of a fraction (>222 ng/ml of 11-3(1)) (Fig. 3A). At lower concentrations (25 or 75 ng/ml of 11-3(1)), binucleated cells were produced and only small patches of actin filaments were observed (Fig. 3B). LC-MS and NMR analyses revealed that the fraction 11-3(1) contained mycalolide A (82% purity) (Supplementary Fig. S1B,C). Mycalolide B and 38-hydroxymycalolide B were also identified in other chromatography fractions, 9-6 and 11-2(1), respectively (Fig. 3 and Supplementary Fig. S1C). These mycalolides have been shown to bind to actin and induce depolymerization *in vitro*^10,11,14^, suggesting that the mycalolides are the major compounds that induce the cellular phenotype. We initially used the fraction 11-3(1) as mycalolide A for live cell imaging (Figs 4–6). However, as the purity of mycalolide A in 11-3(1) fraction was only 82% measured using LC-MS, we prepared a more purified mycalolide A (92% purity) from another *Mycale* sample collected at Kagoshima (Supplementary Fig. S2), by which similar cell phenotypes were observed as for 11-3(1) (Supplementary Fig. S3), and used this for quantitative comparisons with other actin polymerization inhibitors (Figs 7 and 8).

### Visualizing F-actin disrupting activity by live-cell imaging

We investigated the effects of compounds in the fraction 11-3(1) (82% mycalolide A) by live cell microscopy using HeLa cells, which are less motile than MDA-MB-231 cells and suitable for live imaging. Cells were grown on a heated stage in an inverted microscope, and phase contrast images were acquired every 10 min. The fraction 11-3(1) was added to the medium to give a final concentration of 10 ng/ml. Within 10 min of its addition, the morphology of the plasma membrane was altered (Fig. 4A,B), consistent with the inhibition of actin polymerization in lamellipodia. When cells entered into mitosis, chromosome segregation occurred normally and a contractile ring apparently formed. However, most (~90%; 17 out of 19) cells that had entered into mitosis during ~9 hr failed to complete cytokinesis and the outcome was two sister nuclei sharing a single cytoplasm (Fig. 4C,D). This result suggests that mycalolide A in the fraction 11-3(1) inhibits F-actin formation during mitosis, resulting in failure of the completion of cytokinesis.

To directly visualize the effect of the compound(s) in the fraction 11-3(1) on actin filaments in real-time, cells were loaded with rhodamine-actin, and the fluorescence and phase-contrast images were collected every 5 sec. Rhodamine-actin was enriched in cytoskeleton filaments as well as in filopodia and lamellipodia, which were also identified by phase-contrast images. In the normal medium, the actin cytoskeleton appeared quite stable; the steady-state structure can be maintained by the rapid turnover of actin polymerization and depolymerization^21–23^. In contrast, filopodia and lamellipodia moved rapidly during the imaging period for several minutes (Movie 1; Fig. 5A; −7:00 and −4:50). Within a few minutes after the addition of the fraction 11-3(1) at 67 ng/ml, filopodia were retracted and the actin cytoskeleton became disrupted (Fig. 5A; 2:40 and 4:51). Cells then became rounded as a result of losing their cellular tension (Fig. 5A; 10:30).

The disruption of actin filaments was more clearly observed at a higher concentration (300 ng/ml) of the fraction 11-3(1) (Movie 2; Fig. 5B). The disappearance of actin filaments was first observed within a few minutes after addition (Fig. 5B; 0:30 and 1:50). Actin filaments were often disrupted (2:00, closed arrowhead; 2:20, open arrowhead) and retracted with the passage of time. Actin molecules that were released from the cytoskeleton filaments appeared to be mostly diffused throughout the cytoplasm (Fig. 5A; 10:30; and 5B; 10:00) rather than forming aggregations. These observations are consistent with the actin depolymerizing activity of mycalolides^10^, and differ from the effects of actin filament stabilizing agents like amphidinolide H, which has been shown to induce actin aggregations after retraction of the cytoskeleton^20^. Even though the cells became rounder after F-actin disruption, they remained attached to the dish probably through focal adhesion complexes. While the original filopodia disappeared quickly after the addition of the fraction 11-3(1), many spikes reappeared later (Fig. 5A; 10:30; and 5B; 10:00). To examine how such spikes are formed when F-actin is disrupted, we visualized tubulin and F-actin in fixed cells using the specific antibody and phalloidin, respectively. In cells treated with the fraction 11-3(1), spikes contained tubulin but not F-actin (Fig. 6), implying that they are not identical to normal filopodia. We repeated this experiment using mycalolide A that was purified at a higher level (92% purity) from another *Mycale* sp. source, and a similar result was obtained (Supplementary Fig. S3), supporting the view that the active compound that disrupts F-actin in the fraction 11-3(1) is mycalolide A. In addition, when cells were pretreated with nocodazole (to inhibit microtubule polymerization) just before mycalolide A administration, such spikes did not appear (Supplementary Fig. S3). These results suggest that the microtubule filaments can grow to create spikes by pushing out the plasma membrane when the actin cytoskeleton is disrupted, since the extension of microtubules is normally prevented by the actin cytoskeleton.

### Effects of mycalolide A on binucleation

Using the higher (92%)-purified mycalolide A, we compared its effects on cell phenotypes (i.e., binucleation and transcription) with other actin polymerization inhibitors, such as latrunculin B (binding to a nearby ATP binding site of monomeric actin) and cytochalasin D (capping F-actin). To analyze the activity which induces binucleation, two human cell lines, HeLa and MDA-MB-231, were seeded at various densities and treated with the inhibitors for 20 hr. After fixation and staining with Hoechst and phalloidin, the rates of the binucleated cells were counted (Fig. 7A). At the lowest cell density, binucleated cells were produced at high percentages (70–80%) by mycalolide A at 3.6 and 11 nM for HeLa and MDA-MB-231, respectively (Fig. 7B). In the presence of higher concentrations of mycalolide A at the same cell density, few attached cells were observed (indicated by # in Fig. 7B), suggesting that cells had been detached by massive disruption of actin filaments under the influence of excess mycalolide A. When the cell density was increased, the optimized concentration for generating binucleated cells was also increased. Thus, the effects of mycalolide A depended both on the concentration and cell density, probably due to its F-actin severing activity and high-affinity G-actin binding^10^, causing irreversible disruption of the actin filaments. In contrast, effects of cytochalasin D and latrunculin B on binucleation were largely dependent on their concentration and the effective ranges were wider (Fig. 7B). As it has been reported that the effects of cytochalasin are reversible^24^, these molecules may dynamically bind to actin and be less sensitive to the cell density.

### Effect of mycalolide A on transcription

It has been reported that G-actin in the nucleus can control the expression of a subset of genes, partly through the interaction with the myocardin-related transcription factor (MRTF/MAL/MKL1), which is a coactivator of the serum response factor (SRF)^3,4^. The MRTF-SRF complex binds to the serum response elements (SREs) in promoter regions of target genes to facilitate transcription^25,26^. However, when G-actin binds to MRTF, the G-actin-MRTF complex is exported from the nucleus to the cytoplasm, resulting in inhibition of SRF activity^25^. Thus the level of nuclear G-actin influences MRTF-SRF-mediated transcription activation. Consistently, it has been shown that cytochalasin D but not latrunculin B stimulates the SRF-mediated transcription^27^. We then investigated using the luciferase assay whether mycalolide A also affects the transcription from an SRE-containing promoter.

Cells were transiently transfected with the luciferase reporter gene under the influence of the SRE-containing promoter, and treated with different concentrations of actin inhibitors for 3 hr before lysing cells (Fig. 8). We chose a 3 hr time window for inhibitor treatments, because proteins may be synthesized within 3 hr after the induction of transcription^28^ and a longer treatment may induce side effects. In the presence of 100 nM mycalolide A, the luciferase activity was increased (Fig. 8). With a dose of 300 nM mycalolide A, however, no increase in the activity was observed, which is consistent with the narrow optimal range, as seen in the binucleated cell formation (Fig. 4). Control experiments using cytochalasin D and latrunculin B essentially reproduced the previous data^27^. This result suggests that mycalolide A diffuses into the nucleus and outcompetes MRTF for G-actin binding, resulting in increasing G-actin-free MRTF in the nucleus to enhance SRF-dependent transcription. Indeed, MRTF and members of the macrolide family that mycalolide A belongs to (i.e., kabiramide C and jaspisamide A) were reported to bind the cleft between actin subdomains 1 and 3^29,30^. Therefore, mycalolide A may be useful for analyzing G-actin function not only in the cytoplasm but also in the nucleus. In addition to its binding to transcription factors including MRTF, nuclear G-actin is a critical component in multiple complexes for chromatin remodeling and histone modification^31^.

## Conclusions

In this study, we isolated mycalolides from the marine sponge *Mycale* aff. *nullarosette* by using a cell-based assay with DNA and F-actin staining. Live cell imaging then revealed that actin depolymerization caused by the mycalolides results in incomplete cytokinesis that produced binucleated cells. At higher concentrations, mycalolides immediately disrupt actin filaments. Our microscopy-based screening and assay systems have proven useful in measuring biological activities of small molecules essential in cell morphology and molecular organizations, and will be beneficial in discovering future novel compounds.

## Supplementary information

Supplementary Information

Movie 1

Movie 2
