# Supplementary material for: Effect of mycalolides isolated from a marine sponge Mycale aff. nullarosette on actin in living cells
Source: Sci Rep. 2019 May 17;9:7540. doi: 10.1038/s41598-019-44036-2 (PMC6525181; doi:10.1038/s41598-019-44036-2)
Supplement: Supplementary file 1 — Supplementary Information [file 41598_2019_44036_MOESM1_ESM.pdf]

Supplementary information

Effect of mycalolides isolated from a marine sponge *Mycale* aff. *nullarosette* on actin in living cells.

Yoko Hayashi-Takanaka[1,2†], Yuto Kina[3†], Fumiaki Nakamura[3], Shota Yamazaki[4,7], Masahiko Harata[4], Rob W. M. van Soest[5], Hiroshi Kimura[1,6*] and Yoichi Nakao[3*]

[†]These authors contributed equally to this work

[*]To whom correspondence should be addressed

This file includes:

Supplementary Figures S1-S3

Captions for Movies 1 and 2

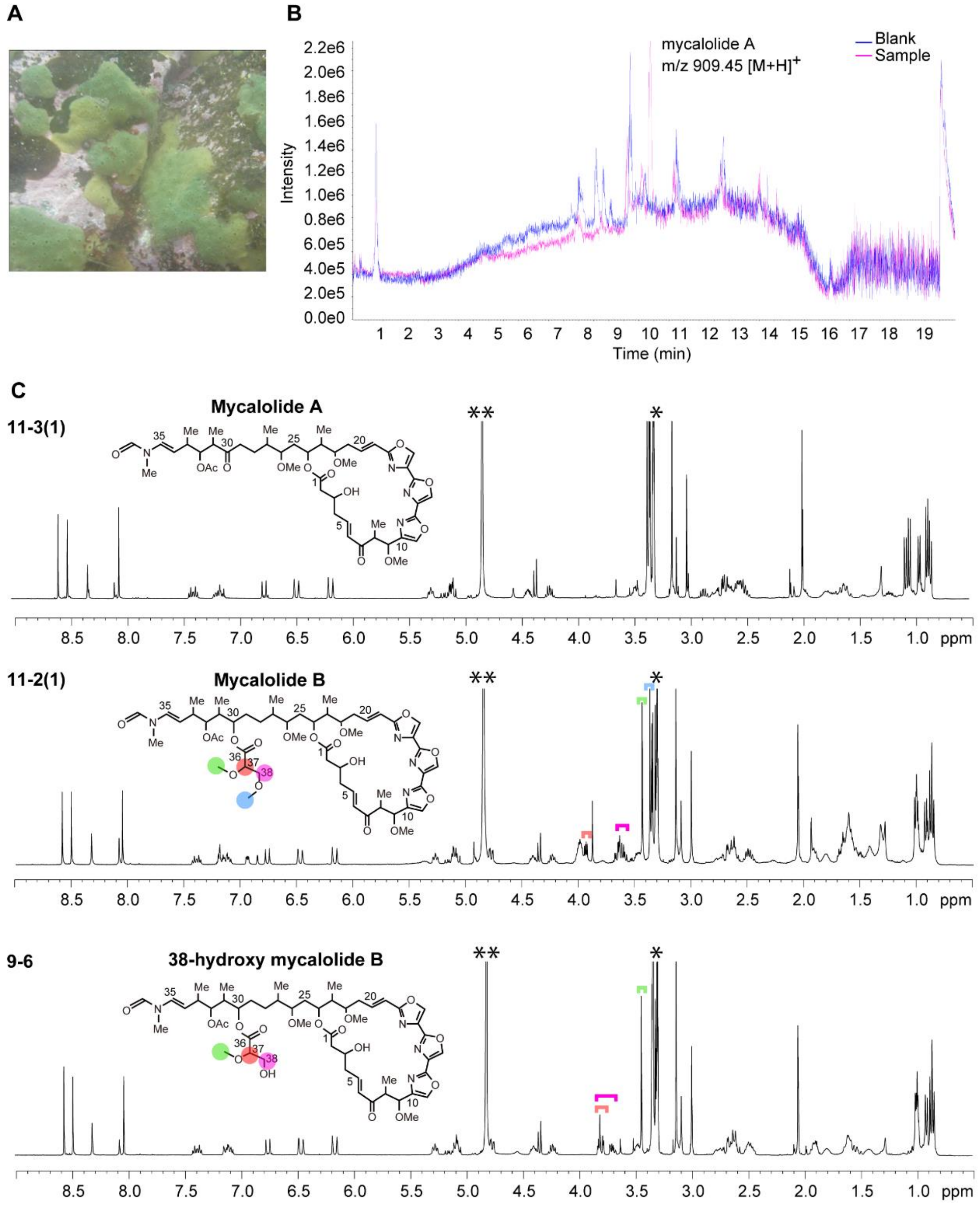

Supplementary Figure S1. ^{1}H NMR and LC-MS spectra of fractions containing mycalolides.

(A) Photograph of marine sponge Mycale aff. *nullarosette* collected at Miyagi prefecture. (B) The total ion chromatogram of the fraction 11-3(1). The purity of mycalolide A (*m/z* 909.45 $[M+H]^+$) was estimated as 82% based on the ion peak area. (C) ^{1}H NMR spectra (CD_3OD, 400 MHz) of fractions 11-3(1), 9-6, and 11-2(1), and the structures of mycalolide A, B, and 38-hydroxymycalolide B. The signals marked with asterisks (* and **) are derived from CHD_2OD and CD_3OH/HDO, respectively. NMR signals corresponding to the protons in the structures are marked.

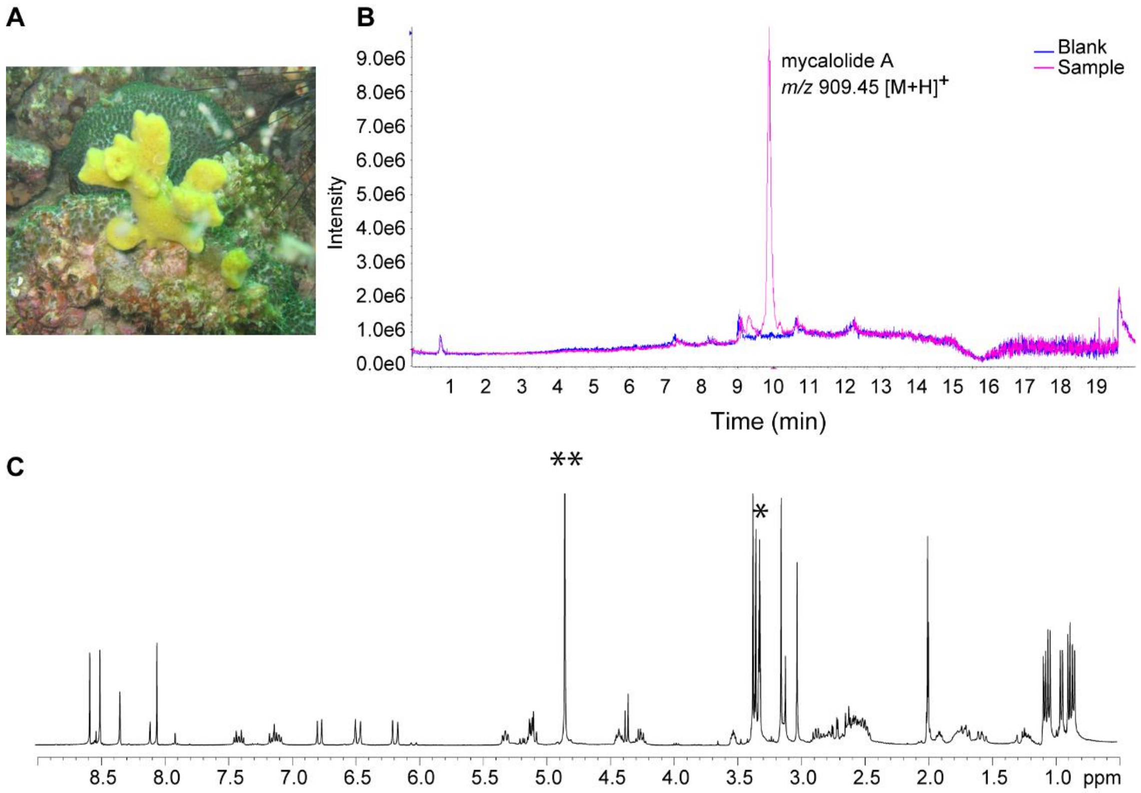

Supplementary Figure S2. ^{1}H NMR spectrum and LC-MS chromatogram of the mycalolide A from Kagoshima *Mycale*.

(A) Photograph of *Mycale* collected at Kagoshima prefecture. (B) The total ion chromatogram. The purity of mycalolide A (*m/z* 909.45 $[M+H]^+$) was estimated as 92% based on the ion peak area. (C) ^{1}H NMR spectrum (CD_3OD; 400 MHz).

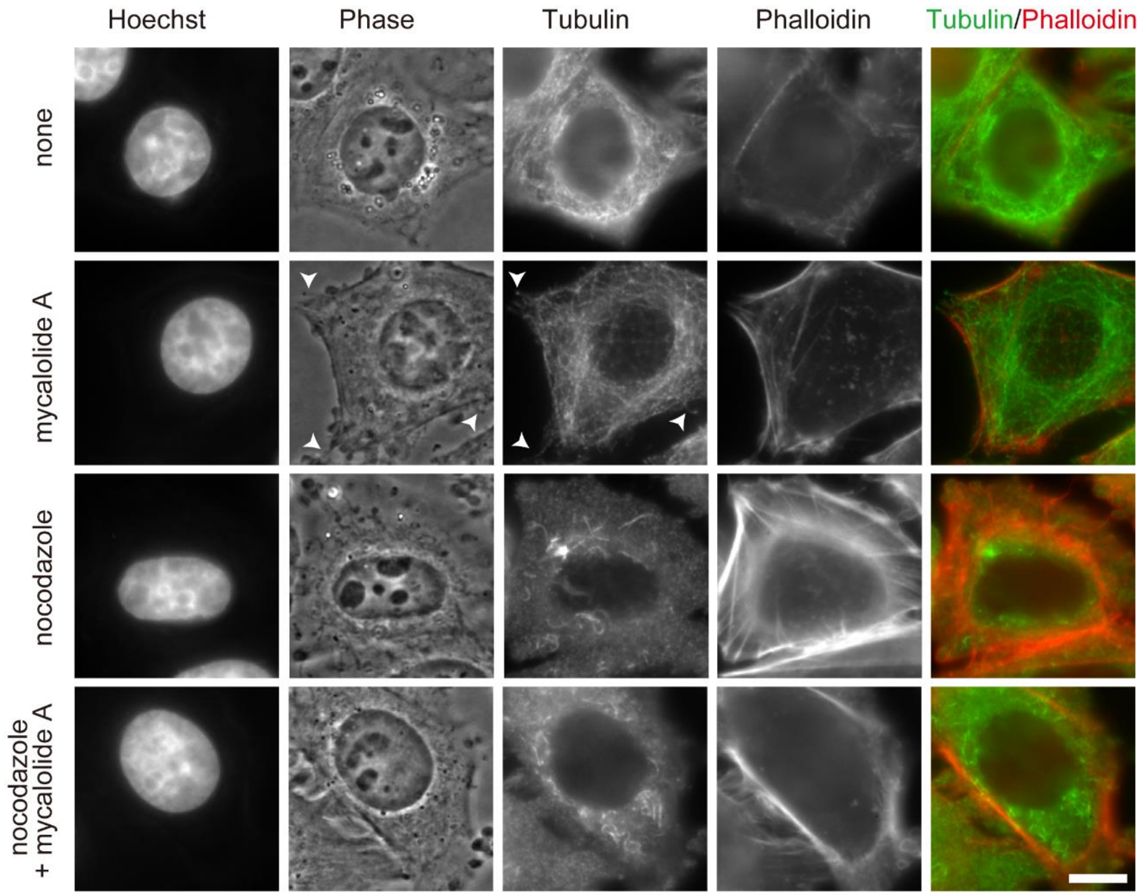

Supplementary Figure S3. Distribution of tubulin in cells treated with mycalolide A and/or nocodazole.

HeLa cells were untreated or treated with 1 μM nocodazole for 1 hr and then administrated with or without 67 nM mycalolide A (92% purity) for 10 min. After fixation, cells were stained with Hoechst 33342, anti-tubulin, and phalloidin. Spikes appeared in mycalolide A-treated cells containing tubulin but not F-actin (arrowheads), unlike filopodia in untreated cells. Bars, 10 μm.

Captions for Movies

Movie 1. Cells treated with the fraction 11-3(1) (82% mycalolide A) at 67 ng/ml. Time-lapse images of rhodamine-actin (middle; red in the merge on the left) and phase contrast (right; gray in the merge on the left) are shown with the elapsed time (h:mm:ss).

Movie 2. Cells treated with the fraction 11-3(1) (82% mycalolide A) at 300 ng/ml. Images of rhodamine-actin (middle; red in the merge on the left) and phase contrast (right; gray in the merge on the left) are shown with the elapsed time (h:mm:ss),
